# Ni/Si-Codoped TiO_2_ Nanostructure Photoanode for Enhanced Photoelectrochemical Water Splitting

**DOI:** 10.3390/ma12244102

**Published:** 2019-12-08

**Authors:** Ting Li, Dongyan Ding

**Affiliations:** Institute of Electronic Materials and Technology, School of Materials Science and Engineering, Shanghai Jiao Tong University, Shanghai 200240, China; litingstar@sjtu.edu.cn

**Keywords:** anodization, doped TiO_2_ nanostructures, photoelectrochemical property

## Abstract

We synthesized Ni/Si-codoped TiO_2_ nanostructures for photoelectrochemical (PEC) water splitting, by electrochemical anodization of Ti-1Ni-5Si alloy foils in ethylene glycol/glycerol solutions containing a small amount of water. The effects of annealing temperature on PEC properties of Ni/Si-codoped TiO_2_ photoanode were investigated. We found that the Ni/Si-codoped TiO_2_ photoanode annealed at 700 °C had an anatase-rutile mixed phase and exhibited the highest photocurrent density of 1.15 mA/cm^2^ at 0 V (vs. Ag/AgCl), corresponding to a photoconversion efficiency of 0.70%, which was superior to Ni-doped and Si-doped TiO_2_. This improvement in PEC water splitting could be attributed to the extended light absorption, faster charge transfer, possibly lower charge recombination, and longer lifetime.

## 1. Introduction

Photoelectrochemical (PEC) water splitting, as a clean and efficient approach for hydrogen production, has attracted extensive attention since Fujishima reported water splitting at TiO_2_ semiconductor photoelectrode [1,2]. As a typical transition metal oxide, TiO_2_ is one of the most suitable photocatalytic materials for water splitting due to its high chemical stability, inexpensive and noncorrosive nature as well as strong catalytic activity [3,4]. However, the practical application of TiO_2_ as a photocatalytic material is limited by its two obvious shortcomings. One is the relatively large intrinsic band gap (3.2 eV for anatase and 3.0 eV for rutile), resulting in an inefficient utilization of solar energy [5]. The other is the high recombination rate of photogenerated electrons and holes in the TiO_2_ nanostructure, which inhibits the migration of photogenerated electrons and holes to the surface of the photocatalyst [6].

In order to solve the above problems, scientists have made continuous efforts to enhance visible-light absorption and/or facilitate the charge separation, like doping metal elements (Zr, Bi, Ru, Ni, etc.) [7,8,9,10,11], doping non-metallic elements (Si, N, P, etc.) [12,13,14], surface decoration [15], and sensitization with dye [16]. Among these methods, doping metal and/or non-metallic elements in TiO_2_ has been extensively investigated. Usually the transitional metals are more likely to be chosen as dopants due to their multiple valence states. The incorporation of transition metal in TiO_2_ creates some defects in the lattice by altering the charge carrier equilibrium concentration. These defects can serve as the shallow trap states in the band gap, which is beneficial to suppressing the charge recombination and thereby enhances PEC activity [17,18]. 

For example, Ni-doped TiO_2_ is considered to be one of the most effective photocatalytic materials and it has been widely studied by various groups. Sharma et al. [19] prepared Ni-doped TiO_2_ through a sol-gel dip coating method and found a strong enhancement of optical absorption and a bandgap narrowing when compared to the undoped TiO_2_. Barmeh et al. [20] fabricated a Ni-doped TiO_2_ thin film using spray coating method and reported that the photocatalytic activity of Ni-doped TiO_2_ was remarkably enhanced since the existence of Ni^2+^ could efficiently suppress the recombination of photogenerated electron-hole pairs on the surface of the photocatalyst. Dong et al. [21] synthesized Ni-doped TiO_2_ through anodizing Ti-Ni alloys and investigated the effect of Ni-doping content on photocatalytic activity. The results showed that Ni-doped TiO_2_ nanotubes fabricated on Ti-1 wt.% Ni alloy exhibited better PEC activity than those on Ti-10 wt.% Ni alloy. In low-Ni doped TiO_2_, Ni 3d orbit states were introduced in the band gap just below the conduction band minimum of TiO_2_. These impurity states could serve as shallow trap centers for photogenerated electrons and holes, and therefore reduced the recombination. Nevertheless, excessive Ni dopants could induce electron-hole recombination centers when serving as deep donors, hindering the generation of photocurrent. In addition, many other transition metal dopants like Zn, Mo, and Fe have been found to be efficient dopants for improved photocatalytic activity of TiO_2_ [22,23,24,25].

However, metal doping in TiO_2_ is complicated by the drawbacks of poor thermal stability, possible photo-corrosion, and expensive ion implantation equipment [26]. From this perspective, non-metal doping has attracted great attention and has been proven to be an effective approach to improve the photocatalytic activity of TiO_2_. Zhou et al. [27] reported that doping of N in the TiO_2_ lattice could broaden the absorption spectrum of TiO_2_, resulting in significantly enhanced optical absorption under visible light at the wavelength less than 500 nm. Although doping of S had a similar effect, it was difficult for S to be incorporated into the TiO_2_ lattice due to its large atomic radius. Among other non-metal promising candidates, the Si element is particularly attractive due to its low cost, nontoxicity, and rich resource. It has been demonstrated that Si doping in TiO_2_ could generate Si-O-Ti bond, which could accelerate the charge transfer and thereby improve the photocatalytic activity [28]. Chen et al. [29] prepared Si-doped TiO_2_ nanorod films through a hydrothermal method, and reported that Si doping improved the hydrophilicity of TiO_2_ by increasing the effective semiconductor/electrolyte interface area, which might account for the improvement in PEC performance. 

Metal/non-metal codoping is another effective approach to improve the photocatalytic characteristics of TiO_2_. Numerous studies have revealed that an appropriate amount of metal/non-metal codoping could achieve effective synergistic effects on photocatalytic activity [30,31]. For example, in V-N doped TiO_2_, the V dopant improved visible light absorption while the N dopant suppressed the recombination of photogenerated electrons and holes. The synergistic effect resulted in a three-time photocatalytic efficiency of V-N doped TiO_2_ compared to the undoped TiO_2_ [31]. Generally, electronic coupling between two dopants is crucial to realize synergistic effects of metal/non-metal codoping [18]. On the other hand, phase interface in TiO_2_ is critically important for the charge transfer process. Recent studies have reported that TiO_2_ with an anatase-rutile mixed phase showed enhanced photocatalytic activity compared to pure anatase (or rutile) phase [3,32]. For the mixed phase TiO_2_, interface defects at the interface of anatase and rutile could serve as charge trapping sites for charge carriers, thereby contributing to a better charge separation. 

Electrochemical anodization of titanium is a relatively simple and facile method to prepare vertically oriented and highly ordered TiO_2_ nanotube arrays, which are considered to be effective structures for PEC activity [33]. Anodization of multicomponent titanium alloy can not only dope foreign elements in TiO_2_ but also modify TiO_2_ nanostructures. In our previous works, we have reported the fabrication of Ni-doped TiO_2_ and Si-doped TiO_2_ [6,12,21], but few works have been reported on the fabrication of Ni/Si-codoped TiO_2_ and the relevant PEC properties. In this work, we successfully fabricated Ni/Si-codoped TiO_2_ nanostructures by anodizing Ti-Ni-Si ternary alloy. The effects of Ni/Si-codoping and annealing temperatures on PEC water splitting properties were investigated. High-efficiency PEC water splitting properties with Ni/Si-codoped TiO_2_ photoanode were achieved. 

## 2. Materials and Methods

As-cast Ti-1Ni-5Si (wt.%) alloy specimens with a dimension of 20 mm × 10 mm × 1 mm were homogenized at 725 °C for 10 h, and then cooled down to room temperature.

Ni/Si-codoped TiO_2_ thin films were synthesized by electrochemical anodization of the alloy samples. Before electrochemical anodization, the alloy plates were mechanically polished with emery papers, followed by ultrasonic cleaning in ethanol and deionized water for 10 min and 20 min, respectively. The electrochemical experiments were carried out in a two-electrode system with the alloy plate as a working anode and Pt foil as a counter electrode. Electrochemical anodization was performed at 40 V for 90 min in a solution of ethylene glycol/glycerol containing 0.30 M (NH_4_)_2_SO_4_ and 0.4 M NH_4_F and 2 vol.% deionized water. All of the experiments were carried out at room temperature. To obtain crystalline phases, as-anodized samples were annealed at elevated temperatures (550 °C, 650 °C, 700 °C, and 750 °C) for 2 h. 

The structural, morphological characterization and crystalline phases of the oxide films were characterized with scanning electron microscope (SEM, FEI SIRION 200, USA) and X-ray diffractometer (XRD, Rigaku Ultima IV, Japan) with a scan range (2θ value) from 10° to 80°. The chemical compositions and chemical states of oxide films were analyzed by energy dispersive spectroscope (EDS, INCA X-ACT, Oxford, UK) and X-ray photoelectron spectroscopy (XPS, AXIS Ultra DLD, Kratos, Japan). The diffuse reflectance absorption spectra of the prepared samples were measured by a UV-visible spectrometer (Lambda 750S, Perkin Elmer Inc., USA) with BaSO_4_ as a reference. 

The PEC measurements were carried out in a three-electrode system with the oxide photoanodes as the working electrodes, Pt foil as the counter electrode and Ag/AgCl as the reference electrode in 1 M KOH (pH = 13.6) solution. All of the photoanodes had an active geometric area of about 1 cm^2^. A 150 W Xe lamp (Lanpu XQ350W, China) was used as an illumination source. The irradiance intensity was controlled at air mass (AM) 1.5 G illumination (100 mW/cm^2^). Linear sweep voltammograms (LSVs), which were characteristic of photocurrent-voltage curves, were measured at a scan rate of 50 mV/s. Electrochemical impedance spectroscopy (EIS) analysis was used to understand the charge transfer process at the interfaces of photoelectrodes and electrolyte. All of the EIS measurements were carried out at the open circuit potential from 10^−1^ Hz to 10^5^ Hz and under AM 1.5 G illumination condition. The Mott–Schottky plots were obtained at a frequency of 1000 Hz in dark conditions.

## 3. Results and Discussion

**Figure 1:**
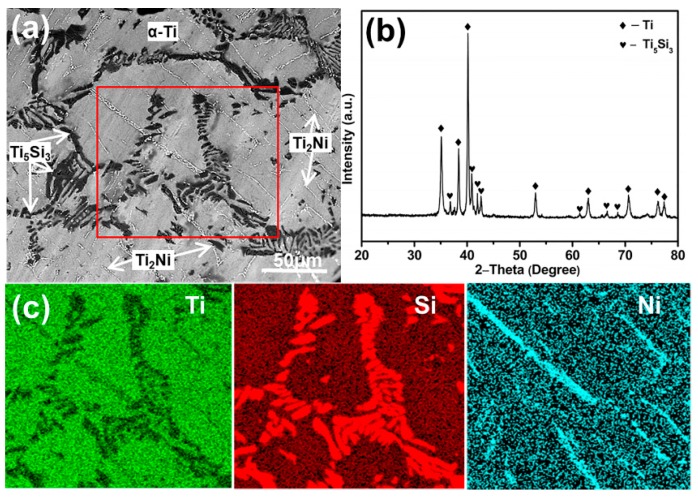
(**a**) Scanning electron microscope (SEM) image and (**b**) X-ray diffractometer (XRD) pattern of the homogenized Ti-1Ni-5Si alloy, (**c**) energy dispersive spectroscope (EDS) element mappings of the marked region in (**a**).

Before anodization, it was necessary to study the microstructure of the ternary alloy substrate. Figure 1a shows a SEM image of the homogenized alloy. Similar to the as-cast alloy reported in our previous work, the gray region, black region, and bright strip-like region were the α-Ti matrix, Ti_5_Si_3_ phase, and Ti_2_Ni phase, respectively [34,35]. The XRD pattern of the alloy (Figure 1b) further proved the existence of the α-Ti and Ti_5_Si_3_ phases [36]. However, the Ti_2_Ni phase was not found due to its low content. Figure 1c shows the EDS element mappings of the marked region in Figure 1a. As expected, Si and Ni elements were rich in the Ti_5_Si_3_ and Ti_2_Ni phase region, respectively.

**Figure 2:**
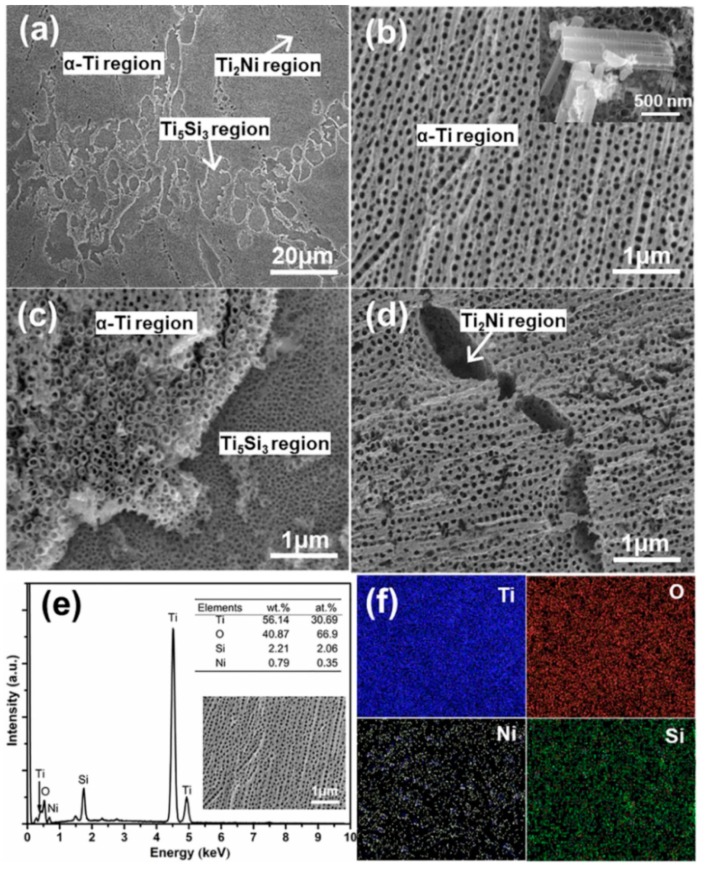
Typical SEM images of Ni/Si-codoped TiO_2_ nanostructures. (**a**) Low-magnification image of the nanostructures, (**b**–**d**) high-magnification images of the nanostructures formed at the α-Ti region, Ti_2_Ni region and Ti_5_Si_3_ region, respectively, (**e**) EDS results of Ni/Si-codoped TiO_2_ layer, (**f**) EDS element mappings of Ti, O, Ni and Si elements for Ni/Si-codoped TiO_2_ layer in (**e**).

Figure 2 shows typical SEM images of Ni/Si-codoped TiO_2_ nanostructures fabricated on the homogenized alloy. According to the phase distributions shown in Figure 1, the α-Ti, Ti_2_Ni, and Ti_5_Si_3_ phase regions were marked in Figure 2a. Apparently, different phases showed different anodization characteristics of surface anodization. Highly ordered nanotubes grew at the α-Ti phase region (Figure 2b). The average outer diameter, wall thickness, and length of the nanotubes were 70, 12, and 1260 nm, respectively. As shown in Figure 2c, the regular nanoporous structure with an average pore diameter of 25 nm was observed at the Ti_5_Si_3_ phase region. However, only corrosion pits were observed at the Ti_2_Ni phase region (Figure 2d). It was known that the electronegativity of Ti, Si, and Ni was in the order of Ni > Si > Ti, supporting that a chemical dissolution rate in the fluoride ions solution was in the order of Ti_2_Ni > Ti_5_Si_3_ > Ti. Therefore, nanopores and etching pits, rather than nanotubes, were observed at the Ti_5_Si_3_ and Ti_2_Ni phase region, respectively. The EDS results (Figure 2e,f) showed the appearance of Ti, Ni, Si and O at the Ni/Si-codoped TiO_2_ layer, demonstrating the successful Ni/Si doping. 

**Figure 3:**
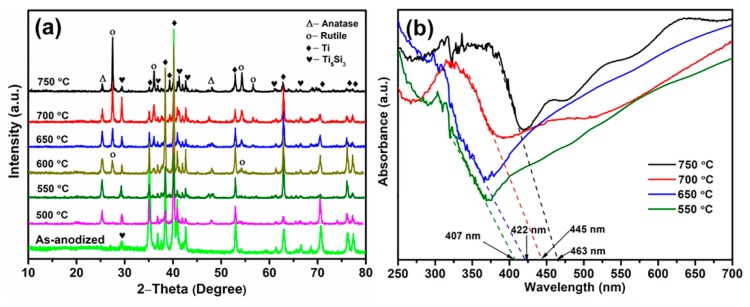
(**a**) XRD patterns (**b**) UV-vis absorption spectra of Ni/Si-codoped TiO_2_ samples annealed at different temperatures.

Figure 3 shows the effects of annealing temperature on the crystalline structure of Ni/Si-codoped TiO_2_ samples. Obviously, the as-anodized samples exhibited an amorphous nanostructure except for the existence of α-Ti phase and Ti_5_Si_3_ phase of the alloy substrate. Therefore, heat treatment at elevated temperatures was necessary to transfer the amorphous nanostructure into a well-crystallized anatase and/or rutile phase. It can be seen that only anatase (101) and (200) peaks at 2θ values of 25.4° and 48.1° (JCPDS card number 21-1272) [37], respectively, were observed for the samples annealed at 500 °C and 550 °C. Rutile (110) and (101) peaks at 27.4° and 54.3° (JCPDS card No. of 21-1276) [38], respectively, began to appear when the annealing temperature increased to 600 °C, demonstrating the starting temperature of phase transformation. It was reported that the starting temperature of phase transformation was 550 °C for Ni-doped TiO_2_ samples [21], suggesting that Si doping in TiO_2_ could suppress the phase transformation from anatase to rutile due to the Si-O-Ti bond, which was consistent with the literature [39,40]. Another study showed that Si-doped TiO_2_ prepared by the anodization method had the same starting temperature of phase transformation (600 °C) [12], but the phase ratio of rutile/anatase in Ni/Si-codoped TiO_2_ was slightly higher than that in Si-doped TiO_2_. This indicated that Ni doping was in favor of facilitating the phase transformation. 

The possible mechanism might be contributed to the high-spin Ni^2+^ [41], similar ion radius of Ni^2+^ (0.072 nm) and Ti^4+^ (0.068 nm) [42], and the linear chain configuration [43]. With a further increase in annealing temperature, anatase peaks gradually diminished in intensity and rutile peaks became dominant. Moreover, no detectable diffraction peaks of Ni-related oxides or Si-related oxides were observed in the XRD patterns, which should be attributed to the doping of Ni and Si atoms into the TiO_2_ lattice by forming the Ni–O–Ti bond [44] and Si–O–Ti bond [45]. This was further confirmed by the XPS analysis. It is no doubt that no characteristic peaks of Ti_2_Ni phase were observed due to the preferential dissolution during anodizing.

The UV-vis absorption spectra of Ni/Si-codoped TiO_2_ samples annealed at different temperatures were employed to investigate the optical properties. As shown in Figure 3b, all samples exhibited a sharp absorption peak near 320 nm, suggesting the existence of an anatase-like TiO_6_^2−^ octahedral phase [46]. The visible light absorption edges were about 407, 422, 445, and 463 nm for the samples annealed at 550, 650, 700, and 750 °C, respectively. The corresponding band gap energies were 3.05, 2.94, 2.78, and 2.68 eV, respectively, which were calculated by the Kubelka–Munk function [3]. In comparison with the pure anatase possessing a band gap of 3.0 eV, the anatase sample annealed at 550 °C exhibited a slight blue shift of absorption edges due to the quantum size effect created by Si doping [47]. Samples annealed at 650, 700, and 750 °C showed enhanced absorption in visible region, which might result from the synergistic effects of anatase-rutile mixed phase and the introduced doping levels serving as shallow trap sites. 

On the one hand, interface defects at the anatase/rutile interface and band alignments increased the non-radiative transition of charge carriers. Also, the carrier mobility/diffusivity and potential barrier in different phases were completely different, which suppressed the charge recombination [32]. On the other hand, doping levels created by hybridization of Si 3p orbital, Ni 3d orbital and O 2p orbits [48] were located in the forbidden gap. Additional photogenerated electronic transitions from the valence band to these doping levels and from these doping levels to the conduction band would result in a remarkable reduction of absorption energy. Compared to Ni/Si-codoped TiO_2_ sample annealed at 650 °C, Ni/Si-codoped TiO_2_ sample annealed at 700 °C exhibited a remarkable red-shift in the visible light range due to its better crystallinity [49]. Furthermore, it had a smaller band gap energy (2.78 eV) than that of Si-doped TiO_2_ (2.82 eV) [12] and Ni-doped TiO_2_ (3.02 eV) [50], which further demonstrated the positive synergistic effects of Si and Ni doping. 

**Figure 4:**
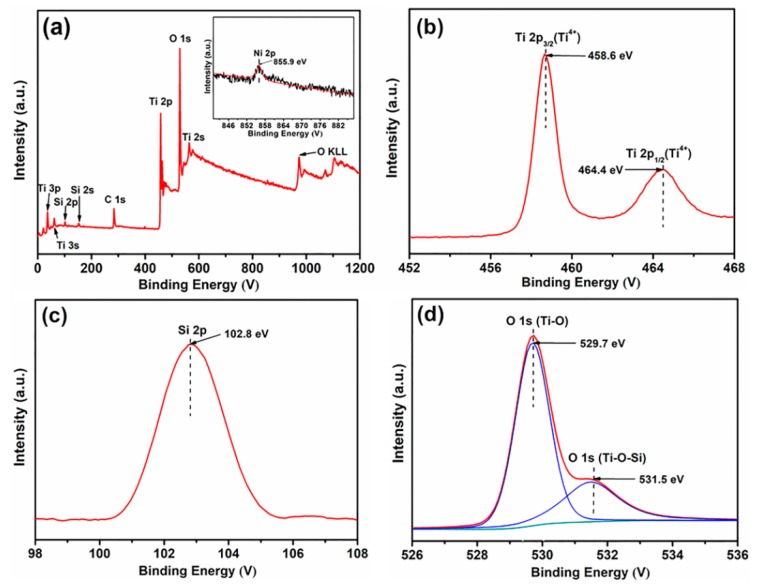
XPS spectra of Ni/Si-codoped TiO_2_ sample annealed at 700 °C. (**a**) Survey spectrum, (**b**) Ti 2p, (**c**) Si 2p, (**d**) O 1s. Inset in (a) shows the Ni 2p spectrum.

Figure 4 shows typical XPS spectra of Ni/Si-codoped TiO_2_ samples annealed at 700 °C. Figure 4a shows the presence of Ti, O, Ni and Si elements on the surface of Ni/Si-codoped TiO_2_. The inset shows that only a weak peak at 855.9 eV was observed in the Ni 2P_3/2_ spectrum of Ni^2+^ ion [11] due to its low doping. As shown in Figure 4b, the peaks at 458.6 eV and 464.4 eV were indexed to Ti 2P_3/2_ and Ti 2P_1/2_ peaks, respectively, indicating the presence of Ti^4+^ state on the nanostructure surface [51]. In Figure 4c, the Si 2p peak at 102.8 eV exhibited a negative shift compared to the Si 2p peak of silica gel (103.3 eV), indicating the formation of Si–O–Ti bonds in the nanostructure and consequently confirming a successful doping of Si in TiO_2_ lattice [52]. The peak at 529.7 eV of O 1s (Figure 4d) indicated the existence of Ti–O–Ti bond, which was consistent with the previous report [28]. In addition, the peak value of 531.5 eV was between 529.7 eV (Ti–O–Ti) and 533.0 eV (Si–O–Si), further confirming the formation of Si–O–Ti bond [53].

**Figure 5:**
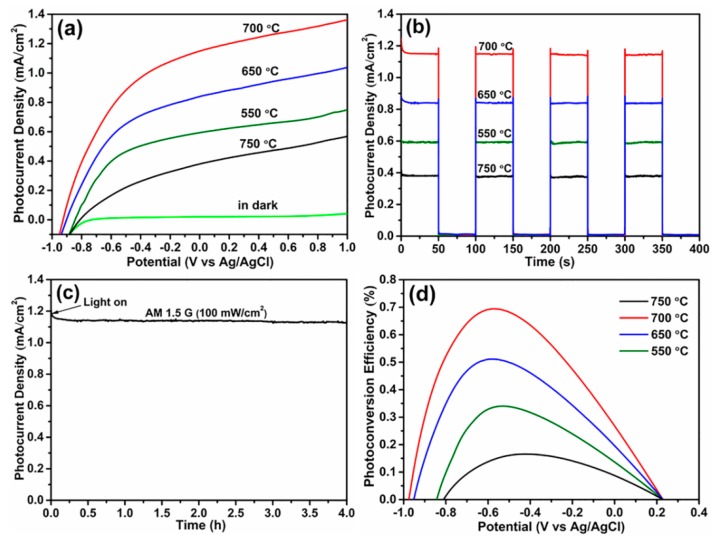
Photoelectrochemical (PEC) water splitting properties of Ni/Si-codoped TiO_2_ photoanodes annealed at different temperatures. (**a**) Linear sweep voltammetry (LSV) curves, (**b**) transient photocurrent density versus time curves, (**c**) photocurrent density versus time curve for photoanodes annealed at 700 °C, and (**d**) photoconversion efficiencies versus applied potential curves.

Figure 5 presents the PEC water splitting properties of Ni/Si-codoped TiO_2_ photoanodes annealed at different temperatures under illumination or in darkness. As shown in Figure 5a, the dark photocurrents of photoanodes could be negligible, implying the absence of drastic PEC water splitting behavior. As can be seen, annealing temperature had an important effect on the water splitting behavior of Ni/Si-codoped TiO_2_ photoanodes. Obviously, the photocurrent density increased with increase of the annealing temperature until 700 °C, after which the LSV curve exhibited a remarkable drop, implying the optimal annealing temperature of 700 °C. Surface defects like oxygen vacancies played a crucial role in PEC water splitting properties. When the annealing temperature was further raised to 750 °C, excessive oxygen vacancies were created in the lattice, which might act as recombination centers for charge carriers and resulted in the reduction of photocurrent density. Among all photoanodes, Ni/Si-codoped TiO_2_ photoanode annealed at 700 °C exhibited the highest photocurrent density of 1.15 mA/cm^2^ at 0 V (vs. Ag/AgCl). This value was about 1.40 times and 1.35 times as high as those of Si-doped TiO_2_ [12] and Ni-doped TiO_2_ [21], respectively, which demonstrated that the Ni/Si codoping significantly enhanced the PEC water splitting.

Figure 5b shows the transient photocurrent response of photoanodes annealed at different temperatures. All photoanodes displayed a rapid photocurrent response. Ni/Si-codoped TiO_2_ photoanode annealed at 700 °C exhibited a photocurrent density of 1.15 mA/cm^2^ at 0 V (vs. Ag/AgCl), higher than those of photoanodes annealed at 550 °C (0.59 mA/cm^2^), 650 °C (0.84 mA/cm^2^), and 750 °C (0.38 mA/cm^2^). This result was in good agreement with the above LSV results. Furthermore, the durability measurement of Ni/Si-codoped TiO_2_ photoanode annealed at 700 °C showed a good thermal stability after 4 hours of AM 1.5 G (100 mW/cm^2^) illumination (Figure 5c).

The photoconversion efficiency *η* can be estimated as [33]:

*η* = *I*(1.23 − *V_RHE_*)/*J_light_* (1)

where *I* is the photocurrent density at 0 V (vs. Ag/AgCl), *J_light_* is the irradiance intensity of 100 mW/cm^2^, and *V_RHE_* is the reversible hydrogen potential, which can be acquired using the expression of *V_RHE_* = *V_Ag/AgCl_* + 0.059 pH +0.1976, where *V_Ag/AgCl_* is the measured potential against Ag/AgCl electrode, and the value of pH is 13.6 (1.0 M KOH aqueous solution). The photoconversion efficiencies of Ni/Si-codoped TiO_2_ photoanodes annealed at different temperatures were presented in Figure 5d. It can be seen that Ni/Si-codoped TiO_2_ photoanode annealed at 700 °C performed a higher photoconversion efficiency of 0.70% at −0.54 V (vs. Ag/AgCl), compared to those of Ni/Si-codoped TiO_2_ photoanodes annealed at 550 °C (0.34% at −0.53 V), 650 °C (0.51% at −0.58 V), and 750 °C (0.17% at −0.43 V). Moreover, this maximum photoconversion efficiency was also higher than those of the reported Si-doped TiO_2_ (0.54%) [12] and Ni-doped TiO_2_ (0.67%) [21], demonstrating a more positive effect of Ni/Si codoping that single Ni or Si doping. 

The intrinsic electronic structures of semiconductors could significantly affect the PEC performance. Mott–Schottky analysis was performed to determine the semiconductor type and obtain the flat band potential as well as donor density of the semiconductor. The Mott–Schottky equation is shown as [54]:

*C*^−2^ = (2/*e*_0_*εε*_0_*N_d_*) [(*V* − *V_FB_*) − *kT*/*e*_0_] (2)

where *C*, *N_d_*, *e*_0_, *ε*, *ε*_0_, *V*, *V_FB_*, *k* and *T* represent the interfacial capacitance, the donor density of n-type semiconductor, the electron charge (1.602 × 10^−19^ C), the dielectric constant of the semiconductor, the vacuum permittivity (8.854 × 10^−12^ F/m), the applied potential bias at the electrode, the flat band potential, the Boltzmann’s constant (1.38 × 10^−23^ J/K), and the absolute temperature, respectively.

**Figure 6:**
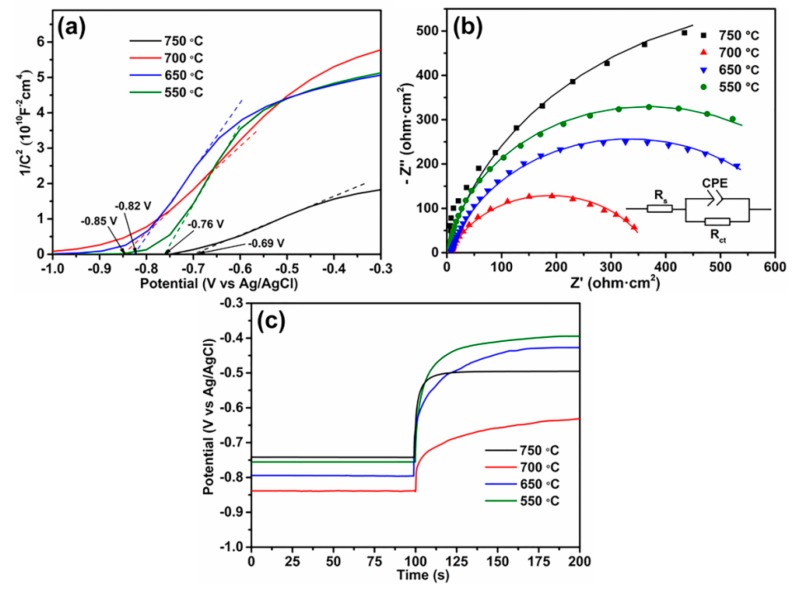
(**a**) Mott–Schottky plots of C^−2^ versus applied potential, (**b**) Nyquist plots, (**c**) transient open circuit potential (OCP) versus time plots for Ni/Si-codoped TiO_2_ photoanodes annealed at different temperatures. The inset in (**b**) shows the relevant proposed equivalent circuit model.

Figure 6a shows Mott–Schottky plots of Ni/Si-codoped TiO_2_ photoanodes annealed at different temperatures. All samples exhibited positive slopes, revealing n-type semiconductor as expected. The *V_FB_* values were calculated from the extrapolation of the line to 1/*C*^2^ = 0 [55]. The Ni/Si-codoped TiO_2_ photoanode annealed at 700 °C, exhibited a more negative *V_FB_* value (−0.85 V), suggesting a larger driving force to charge separation and consequently a more effective charge separation [56].

Meanwhile, the donor density *N_d_* can be determined from the linear slopes in Mott–Schottky plots, according to the following equation:

*N_d_* = (2/*εε*_0_*e*_0_) [d(1/*C*^2^)/d*V*]^−1^ (3)

where *e*_0_ = 1.602 × 10^−19^ C, *ε* is the dielectric constant of the semiconductor (60 has been assumed for the oxide layer on Ti [57]), *ε*_0_ = 8.854 × 10^−12^ F/m, and d(1*/C*^2^)/d*V* is the positive liner slope in the plots. Usually a smaller positive liner slope means a higher donor density. The calculated *N_d_* values of the photoanodes annealed at 550, 650, 700, and 750 °C were 1.14 × 10^19^, 1.30 × 10^19^, 1.83 × 10^19^, and 4.12 × 10^19^ cm^−3^, respectively. Although the Ni/Si-codoped TiO_2_ photoanode annealed at 750 °C had a high concentration of donor density, which could improve the conductivity of the photoanodes, its excessive oxygen vacancies in the lattice might instead act as the recombination centers for the photogenerated electron and holes and thus harm PEC water splitting [58]. 

Electrochemical impedance spectra (EIS) were performed on Ni/Si-codoped TiO_2_ photoanodes annealed at different temperatures to better understand the charge transfer process at the photoelectrode/electrolyte interface, and the corresponding Nyquist plots are shown in Figure 6b. The inset shows the relevant proposed equivalent circuit model, where R_s_ stands for the resistance of electrolyte, R_ct_ for a charge-transfer resistance of photoanode/electrolyte interfaces, and R_CPE_ for the capacitance phase element [59]. In general, the semicircle diameter is characteristic of the interface charge transfer resistance (R_ct_). Obviously, the Ni/Si-codoped TiO_2_ photoanode annealed at 700 °C featured the smallest diameter than other photoanodes. As illustrated in the fitting of solid curves in Figure 6b, the estimated R_ct_ value for the Ni/Si-codoped TiO_2_ photoanode annealed at 700 °C was 364 Ω. This value was smaller than those of the photoanodes annealed at 550 °C (728 Ω), 650 °C (649 Ω), and 750 °C (1347 Ω). This suggests a faster charge transfer rate at the photoelectrode/electrolyte interface of Ni/Si-codoped TiO_2_ photoanode annealed at 700 °C, therefore enhancing the PEC properties [12,21,60,61]. These results were well consistent with the results of the Mott–Schottky measurements.

To investigate the charge recombination kinetics of Ni/Si-codoped TiO_2_ photoanodes, transient open circuit potential (*V_OC_*) versus time plots were recorded upon turning off the illumination. As shown in Figure 6c, negative open circuit potential (OCP) values appeared upon illumination, as the accumulated photoelectrons in Ni/Si-codoped TiO_2_ photoanodes made the Fermi level a negative shift. When turning off the illumination, the decay in *V_OC_* occurred due to the charge recombination. A lower decay rate of *V_OC_* indicated slower recombination kinetics. The lifetime of photoelectrons (τ) before combination is crucial for the PEC properties. It can be estimated by the following equation [33]:

τ = −(*kT*/*e*) (d*V_OC_*/d*t*)^−1^ (4)

where *k*, *T*, and *e* are the Boltzmann’s constant, the absolute temperature, and elementary charge, respectively. Clearly, the Ni/Si-codoped TiO_2_ photoanode annealed at 700 °C exhibited the lowest decay rate of *V_OC_* than other photoanodes, indicating slower recombination kinetics and a longer lifetime. These results explain why the Ni/Si-codoped TiO_2_ photoanode annealed at 700 °C presented better PEC water splitting. 

**Figure 7:**
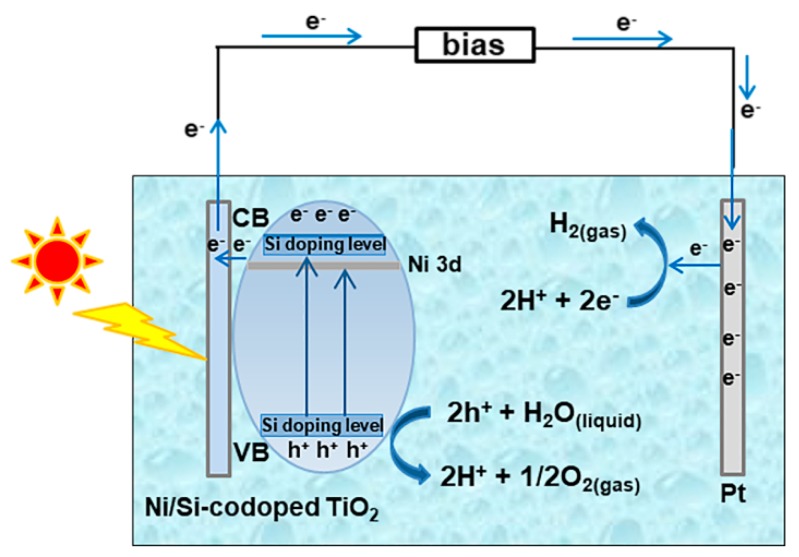
Proposed mechanism of PEC water splitting on Ni/Si-codoped TiO_2_ photoanode.

Figure 7 illustrates the possible mechanism of PEC water splitting behavior on Ni/Si-codoped TiO_2_ photoanodes. In Ni/Si-codoped TiO_2_, Ni and Si codoping contributed to an extended visible light absorption region and a narrower band gap, as analyzed in UV-vis absorption results. It was reported that after a low Si-doping, the valence band of TiO_2_ became broadened through hybridizing Si 3s and O 2p states, and the conduction band became broadened through hybridizing Si 3s, Si 3p and Ti 3d states, resulting in a narrowing band gap. Meanwhile, Ni 3p doping levels were introduced in the band gap just below the conduction band minimum of TiO_2_. These hybrid valance/conduction bands with large dispersion as well as Ni doping levels benefited the mobility of photogenerated electrons and holes. Once illuminated, photogenerated holes (h^+^) could quickly oxidize H_2_O to O_2_ and H^+^ (2h^+^ + H_2_O_(liquid)_ → 2H^+^ + 1/2O_2(gas)_). Simultaneously, photogenerated electrons (e^−^) could effectively transport to the cathode (Pt) through the external circuit and involved the reduction of H^+^ to H_2_ (2H^+^ + 2e^−^ → H_2(gas)_), consequently enhancing PEC water splitting. Based on the principles of PEC water splitting reactions, attempts towards reducing the recombination rate of photogenerated electron-hole pairs have been made. Woo et al. [62] prepared N-doped TiO_2_ microporous structure with an anatase-rutile mixed phase through micro-arc oxidation method and used as photoanode for hydrogen generation. Their study showed that N doping in TiO_2_ and the increase of rutile content led to more efficient charge separation compared to undoped TiO_2_, but the maximum photoconversion efficiency (0.4%) was still lower than that of Ni/Si-codoped TiO_2_ photoanodes reported here. Choi et al. [63] prepared highly ordered TiO_2_ nanotube arrays through a two-step electrochemical anodization. They reported that the loading of Au nanoparticles onto the nanotube arrays remarkably increased the photocurrent density up to 1.67 times. Liang et al. [64] decorated hydrogenation-treated TiO_2_ with carbon quantum dots (CQDs). The highest photocurrent density of the composite was six times higher than that of pristine TiO_2_. This can be explained in that hydrogenation treatment caused the formation of Ti^3+^ species and oxygen vacancies, which could act as shallow donors to suppress the charge recombination. On the other hand, the decorated CQDs could act as electron reservoirs to trap photogenerated electrons, thereby reducing the charge recombination rate. All of these studies provide inspiration for designing modified Ni/Si-coped TiO_2_ for high-efficient PEC water splitting performance.

## 4. Conclusions

In this work, we fabricated Ni/Si-codoped TiO_2_ nanostructures through anodizing Ti-1Ni-5Si alloy substrates in ethylene glycol/glycerol solutions containing water. It was found that Ni and Si elements were successfully doped in the TiO_2_ nanostructure. The annealing temperature had a great effect on the PEC properties of Ni/Si-codoped TiO_2_ photoanodes. The Ni/Si-codoped TiO_2_ photoanode annealed at 700 °C exhibited the best PEC water splitting properties. The highest photocurrent density was about 1.15 mA/cm^2^ at 0 V (vs. Ag/AgCl), corresponding to a photoconversion efficiency of 0.70%, which was higher than those of Ni-doped TiO_2_ and Si-doped TiO_2_. The extended visible light absorption range, faster charge transfer, and possibly lower charge recombination as well as longer lifetime were responsible for the high efficient PEC water splitting.
